# Unveiling the Reactivity of Fluoropolymers with Sodium Metal: Mechanistic Insights and Battery Implications

**DOI:** 10.1021/jacsau.5c00552

**Published:** 2025-06-26

**Authors:** Cheng-Tien Hsieh, Wenda Wu, Karam Eeso, Zhitao Chen, Johannes Leisen, Alexandros Filippas, Michelle Lehmann, Guang Yang, Nian Liu

**Affiliations:** † School of Chemical and Biomolecular Engineering, 1372 Georgia Institute of Technology, Atlanta, Georgia 30332, United States; ‡ Chemical Science Division, 6146 Oak Ridge National Laboratory, Oak Ridge, Tennessee 37831, United States; § School of Chemistry and Biochemistry, Georgia Institute of Technology, Atlanta, Georgia 30332, United States

**Keywords:** Poly(vinylidene fluoride-*co*-hexafluoropropylene) (PVDF-HFP), Polyvinylidene fluoride (PVDF), Polytetrafluoroethylene (PTFE), Interfacial side reactions, Sodium (Na)-based battery

## Abstract

Poly­(vinylidene fluoride-*co*-hexafluoropropylene) (PVDF-HFP) has broad applications across various metal-ion battery systems, such as a binder for electrodes, a supporting matrix for electrolytes, and a separator material. Due to its excellent mechanical properties, PVDF-HFP has become an excellent candidate for fabricating gel and solid-state electrolytes in sodium-based batteries. However, in this study, we noticed notable side reactions occurring at the interface of PVDF-HFP membranes and Na metal. These reactions not only alter chemical compositions but also further affect the surface morphology and adhesive properties of membranes. Similar phenomena are observed in other polyfluoroalkyl-based membranes (PVDF and PTFE). Therefore, we systematically studied the reaction mechanisms between the Na metal and these polymers. The influence of different functional groups (−F, −CF_3_, −H) and their arrangement on the reaction extent has also been discussed. Finally, we concluded with the key factors driving these side reactions and provided new perspectives for designing polymers tailored for sodium-based batteries.

## Graphical abstract

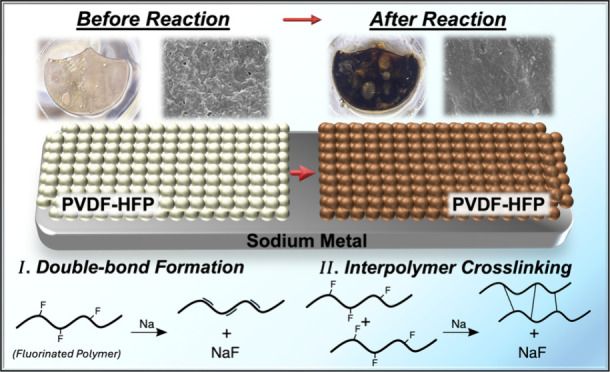

## Introduction

Sodium (Na), due to its abundant availability and low cost, has become a promising alternative to address and mitigate the dramatically increasing costs of lithium (Li) and its associated active materials, such as cobalt.^1^ Utilizing earth-abundant materials in energy storage solutions offer a sustainable and cost-effective path for enhancing grid resilience and power storage. These scalable technologies not only ensure long-term energy security but also drive the forward-thinking integration of energy sources into a power infrastructure. However, although Na and Li both belong to the alkali metal group and share the most similar properties among metallic elements, Na has lower cohesive energy (CE_Na_ = 107 kJ/mol) and first ionization energy (IE_Na_^first^ = 495.8 kJ/mol) than Li (CE_Li_ = 158 kJ/mol; IE_Li_^first^ = 520.2 kJ/mol), making it more prone to donating electrons.^2^ Consequently, Na metal more readily facilitates radical formation and is more favorable to participate in radical-based reactions. Even though Li has a more negative redox potential (*E*_Li_^0^ = −3.04 V vs SHE) than Na (*E*_Na_^0^ = −2.71 V vs SHE), the redox potential reflects not only cohesive and ionization energies but also solvation energy, making it an indirect indicator of the reactivity of alkali metals toward specific functional groups. Therefore, to determine the feasibility of transferring and applying Li-relevant knowledge to Na battery systems, it is necessary to first understand the differences in chemical and electrochemical performance of these two metals.

Poly­(vinylidene fluoride-*co*-hexafluoropropylene) (PVDF-HFP) and polyvinylidene fluoride (PVDF) are commonly used polymers in various kinds of metal-ion batteries, such as lithium-ion, sodium-ion, and magnesium-ion batteries.^3,4^ They exhibit outstanding electrochemical and chemical stability, leading to long-term cycling performance with relatively low or even no capacity fades. Additionally, their excellent mechanical properties are attractive. Their applications have extended from just being utilized as a binder on electrodes to functioning as separator materials or supporting matrixes for fabricating gel and solid-state electrolytes.^5–7^ Due to their low cost and highly tunable porosity and mechanical strength, we planned to replace the expensive ion-exchange membranes in Na||S nonaqueous redox-flow batteries with easily fabricated PVDF-HFP membranes, thereby further reducing costs for grid-scale energy storage.^8,9^ In this study, porous membranes were fabricated using the phase inversion method with a water content of 4.2 wt %, as it showed the best electrochemical performance among three different solvent compositions (Figure S1). However, despite PVDF-HFP being widely utilized in various electrolytes for Na-based batteries,^10,11^ we observed abnormal cycling fluctuations and a gradually increased overpotential and internal resistance during the preliminary cell tests (Figures S2 and S3). Interestingly, the cycled membranes had significant morphology changes only on the side in direct contact with Na metal (Figure S4). These phenomena prompted us to understand the potential side reactions at the interface between PVDF-HFP and the Na metal. Prior to our study, no research had explored the stability of PVDF-HFP in Na-based systems. These degradation reactions could negatively impact the electrochemical performance of batteries by altering the microporous, semicrystalline, and polar structure, thereby decreasing the Na^+^ transport efficiency throughout the cycling process.^12,13^ The most relevant study was conducted by Qian et al., which examined PVDF as a cathode binder and pointed out its instability in sodium and potassium ion batteries.^14^ Yet, the proposed mechanisms were hypothetical without any experimental evidence. This gap in understanding highlights the necessity for a comprehensive investigation of the interactions between fluorinated polymers and the Na metal.

In this study, we elucidate the defluorination mechanisms of polyfluoroalkyl substrates (PFAS), including PVDF-HFP, PVDF, and poly­(tetrafluoroethylene) (PTFE), with Na metal through a combination of experimental characterizations and computational simulations. We reveal that the fluoro groups on the polymer backbone react with Na metal, causing color changes and cross-linking within membranes. Furthermore, experimental results and density functional theory (DFT) calculations demonstrate that the reaction rate depends on the fluorination percentage and the chain structure with other functional groups (−H and −CF_3_). Overall, this work systematically investigates the underlying principles governing the reactivity of different polyfluoroalkyl polymers with Na metal, offering valuable insights into designing new functional polymers in Na-based batteries.

## Results and Discussion

### Changes of the PVDF-HFP Membrane after Reaction with Sodium Metal

We noticed that PVDF-HFP membrane’s color changed from white (transparent in the solvent) to brown upon contact with Na metal (Figure S5a). To identify the key factors contributing to this phenomenon, a series of experiments were conducted to examine the interactions among PVDF-HFP, Na metal, and Na-based electrolytes. First, PVDF-HFP membranes were soaked in tetraethylene glycol dimethyl ether (TEGDME) electrolytes with various concentrations of NaClO_4_, and no obvious changes were observed between the soaked and pristine membranes after 14 days (Figure S5b). The SEM images in Figures S6a and S6b further confirmed that, except for tiny physical variations in volume swelling and shrinkage, their surface morphologies remain the same. Second, Na metal was put in 1.0 M NaClO_4_ electrolytes, and no brown-colored byproducts were generated (Figure S5c). These results indicate that the dark coloration comes specifically from the byproducts originating during the reactions between the PVDF-HFP membrane and the Na metal and is independent of Na^+^ and the solvent. However, the solvent plays a vital role as a medium for reactions to happen (Figure S5d). Thus, we pressed PVDF-HFP and Na metal together and immersed them in the TEGDME solvent to monitor color changes. The membrane progressively darkened with an increased reaction time (Figures 1a and S5e). SEM analysis (Figures 1b and S6d) also revealed that these side reactions blocked microscopic pores, reducing membrane porosity and leading to a flatter structure. Beyond the influence of time, electrochemical cycling further accelerated these reactions.

**Figure 1:**
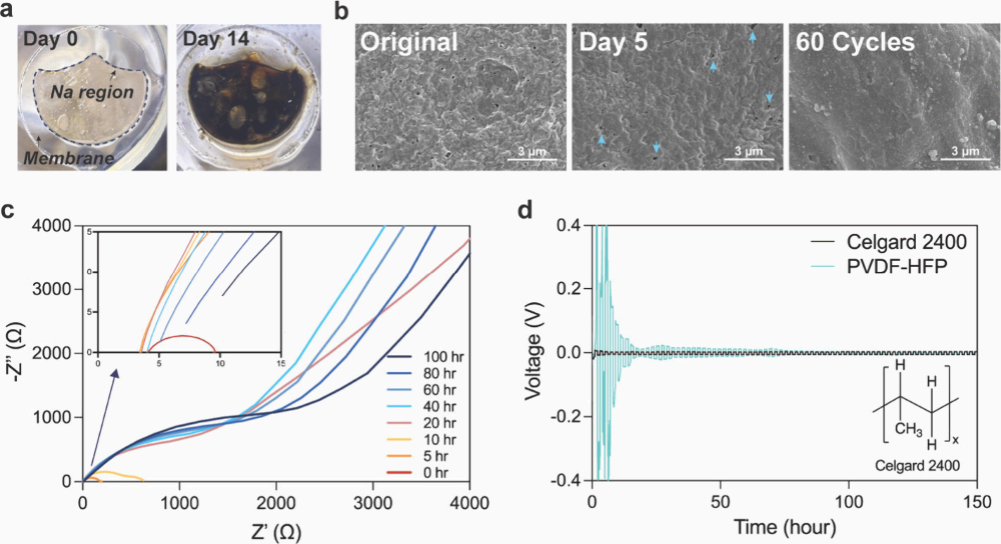
(a) Photographs of the PVDF-HFP membranes with Na metal in TEGDME solvent at different reaction times. (b) SEM images of PVDF-HFP before and after the reaction. From left to right: Before reacting with Na; after reacting with Na for 5 days in TEGDME (arrows highlight the residual holes on the membrane); and after cycling for 60 rounds in 1.0 M NaClO_4_ electrolyte. The reaction time of 60 cycles is equal to 5 days. (c) Nyquist plot of the PVDF-HFP membrane in Na||Na symmetric cells with different resting times without cycling. Inset: Magnified view of the same figure, highlighting the ohmic resistance of each condition. (d) The cycling performance (±0.1 mA) of Na||Na symmetric cells with PVDF-HFP and Celgard 2400 membranes and the chemical structure of Celgard 2400 (polypropylene).

Moreover, these side reactions negatively impact the internal resistance of the coin cell (Figure 1c and Tables S3 and S4). Ohmic and charge-transfer resistances gradually increased with reaction time owing to the formation of the solid electrolyte interphase (SEI) layer on the Na metal and the morphological alternations in the PVDF-HFP membrane. In Figure 1d, by comparing the cycling performance of the PVDF-HFP membrane with a nonfluorinated Celgard 2400 membrane in the Na||Na symmetric cell, we found that the polypropylene-composed membrane was relatively stable in the Na metal environment. Therefore, we suggest that the degradation reactions are specifically associated with the fluorinated nature of the PVDF-HFP polymer.

For the sake of obtaining a full picture of the reaction mechanisms and identifying the byproducts of the side reactions, further surface characterizations are needed. Figure 2a presents PVDF-HFP membranes’ FT-IR spectra at different reaction times. Before reacting with Na metal, C–F-related peaks on the pristine PVDF-HFP membrane were stronger than other signals.^15–17^ As the reaction progresses, the C–F peaks gradually diminish, and some new peaks are generated. In the orange region, the ratio of the C–F bond (∼1180 cm^–1^) to the C–CF_3_ bond (∼1070 cm^–1^) decreased dramatically (Table S5). Due to the intrinsic chemical stability of the trifluoromethyl group, the fluorine atoms bonded to it are significantly more stable than those attached directly to the carbon backbone. Thus, the fluoro groups on the carbon backbone were the primary reactants rather than those in the trifluoromethyl group. A similar trend is also observed in the red region, where the ratio of the C–H peak (∼760 cm^–1^) to other C–F peaks (∼790 cm^–1^ and ∼870 cm^–1^) increased. In the yellow region, the C–F signal (∼490 cm^–1^) weakens substantially, while a new peak (∼400 cm^–1^) related to NaF or some Na-based species emerged, further confirming that the reactions are between Na and fluoride.^18^ Furthermore, the peaks that appeared within the blue region are associated with C=C (∼1580 cm^–1^) and conjugated C=C (∼1650 cm^–1^) bonds. The formation of the conjugated C=C bond explains why the color of films became darker after reaction.^19–21^ Last, the intensity of C–H-related signals in the green region increases due to the reduction in the intensity of other spectral peaks.

**Figure 2:**
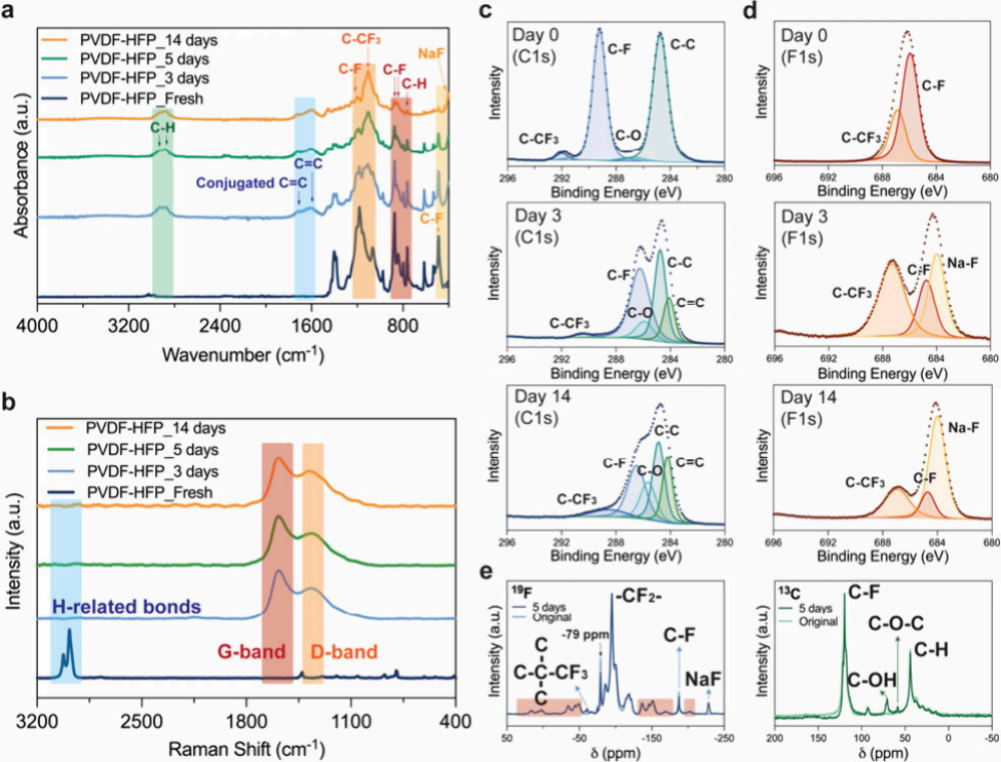
Surface elemental analyses of the PVDF-HFP membranes at varying reaction times with Na metal. (a) Normalized FT-IR spectra. (b) Normalized Raman spectra. (c) XPS data for C 1s. (d) The XPS data for F 1s. (e) Normalized ^19^F and ^13^C MAS NMR spectra (24 kHz). The peaks inside the red region in the ^19^F spectra belong to spinning sidebands (SSBs), not real peaks.

Figure 2b shows the Raman spectra of PVDF-HFP at different reaction times. The peaks in the red and orange areas correspond to the graphene band (G-band) and the disorder band (D-band), respectively.^22^ As the reaction degree increases, the G-band/D-band ratio decreases, showing an increase in the level of structural disorder (Table S6). Apart from graphene-related signals, no Raman signals related to alkyne linkages were detected.^23,24^ Meanwhile, all C–H bond signals in the blue region disappeared upon the membrane reacting with Na, which is due to the high sensitivity of Raman analysis to nonpolar bonds in the molecular structure as the signal intensities in other regions were much higher in comparison.^25^

Next, to determine the elemental composition of byproducts on the membrane surface, we performed XPS analysis, and the Gaussian-fitting results are presented in Figures 2c, 2d, and S7 and Table S8.^26^ In the C 1s spectrum, the C–F bond signal (289.2 eV) decreased, while a new peak related to C = C (284.2 eV) emerged simultaneously after the reaction.^27^ Following the reaction, the C–CF_3_ peak (291.9 eV) shifted to a lower binding energy region because some fluorine atoms originally surrounding it were replaced by carbon atoms through cross-linking. This substitution decreases its binding energy as the high electronegativity of fluorine would induce significant electron withdrawal effects from adjacent carbon atoms. A downward shift in the overall peak position of the C 1s spectrum was also due to the reducing fluorine concentration. In the F 1s spectrum, the intensity of the C–F peak (685.9 eV) decreased sharply and became distinguishable from the C–CF_3_ peak (686.8 eV). This conversion occurred because fluoro groups reacted with Na to form NaF compounds (683.9 eV), which caused peak shifting.^28^ When the reaction extent intensified over time, stronger NaF and C=C bond signals and weaker intensity for the C–F bonds were observed.

Finally, NMR spectroscopy was employed to provide additional evidence confirming the chemical changes arising within the PVDF-HFP membranes. Solid-state MAS ^19^F, ^13^C, and ^1^H NMR results are exhibited in Figures 2e and S9. Due to strong dipolar couplings between ^19^F sites, the spectra contained multiple spinning sidebands (SSBs). Therefore, isotropic peaks at characteristic chemical shift values were identified via recording spectra at different spinning speeds (Figure S8). In the ^19^F spectrum, peaks corresponding to −CF_3_ groups attached on a quaternary carbon (−62 ppm), and fluorides in NaF (−229 ppm) appeared after reacting.^27,28^ The former is direct evidence of interpolymer cross-linking, indicating that the fluorine atom originally bonded to the central carbon was replaced by a carbon atom from another polymer chain. Moreover, the peak at −79 ppm represents the crystallinity-to-amorphous ratio in the polymer, the polar extent of the C–F groups. The intensity of this peak decreased postreaction, meaning the generation of more asymmetric C–F bonds within the membrane. Since the magnetization transfers more efficiently to the crystallite region than to the amorphous domains, the intensity of this peak can be further lowered if the extent of polarity is enhanced.^29^ The ^13^C spectrum showed a pattern dominated by fluoride-bonded carbons (120 ppm) and proton-bonded carbons (44 ppm).^30^ It is worth noting that the signal of ^13^C=C bonds (115–140 ppm for alkene carbons) might be overlapped by the ^13^C–F signal due to their similar chemical shifts.^31^ The new peaks at 57 and 70 ppm cannot be unambiguously assigned to specific functional groups. However, hydroxyl-bonded carbon (C–OH) and ether carbon (C–O–C) are plausible candidates, potentially generated during the ethanol cleaning process before the solid-state NMR measurements.^32^

### Comparison between PVDF-HFP, PVDF, and PTFE: The Influence of Different Functional Groups

Following our study on PVDF-HFP membranes, we aim to further unravel the fundamental principles of these degradation mechanisms by testing other polyfluoroalkyl-based materials in interaction with Na metal. PVDF and PTFE were selected because they have similar chemical structures to PVDF-HFP and are also widely used in Na-based batteries.^33,34^ Unlike PVDF-HFP, PVDF and PTFE lack trifluoromethyl groups and have different fluoride-to-hydrocarbon ratios in their compositions (Figure 3a). These structural differences result in distinct color change trends during the reaction process. As seen in Figures 3b and S10, the color of PVDF membranes changed from transparent to light brown within five min of contact with Na metal. However, the chromatic response appeared to cease or slow down over prolonged contact time. In contrast, the PTFE membrane had no color change upon contact with Na metal in a short amount of time but turned deep black after 1 day (Figure S10c). Over time, these differences became more significant. The underlying reason for the dramatic color change of PVDF upon initial contact with Na but no further noticeable changes for a longer reaction time remains unclear. One possible explanation is that PVDF polymer consists of 50% methylene hydrocarbon moieties, which greatly reduce the electrophilicity of the polymer chain and make it less reactive toward electron-rich Na metal. In addition, the slower initial reaction of PTFE can be attributed to its poor wettability with the TEGDME solvent. In their SEM images (Figure 3c), the surface morphology transition trends were similar to those on the PVDF-HFP membranes. After reactions, the pore size of PVDF was visibly reduced, and NaF particles were generated on its surface. Although NaF molecules were also detected on the surface of the PTFE membrane, the fiber-like structure of PTFE made it hard to check the pore size change before and after the experiment. Yet, the extremely brittle physical strength and serious shape deformation of reacted PTFE samples indicate severe decomposition happened and can serve as additional evidence of cross-linking (Figure S10d).^35^ These observations verify that both PVDF and PTFE membranes reacted with Na metal as well.

**Figure 3:**
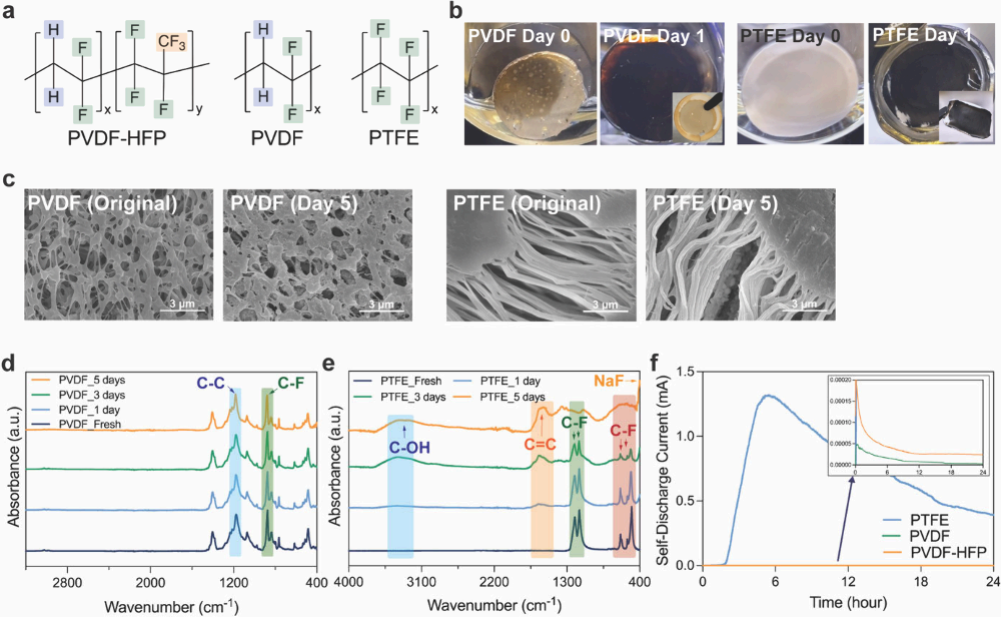
(a) Chemical structures of PVDF-HFP, PVDF, and PTFE polymers. (b) The photographs of the PVDF or PTFE membranes at different reaction times with Na metal in TEGDME. Inset: The picture of films after drying. (c) SEM images of PVDF and PTFE membranes before and after reaction. (d) Normalized FT-IR data of PVDF at different reaction times. (e) Normalized FT-IR data of PTFE at different reaction times. (f) Chromatometry data of different polymers. Inset: Magnified view of the same figure, highlighting the PVDF-HFP and PVDF self-discharge current section

Similarly, FT-IR spectroscopy was conducted to inspect the decomposition products. As shown in the IR spectra of PVDF (Figure 3d), negligible changes were noted after the reactions. The only difference was an increase in the ratio of the C–C bond (∼1185 cm^–1^) to the C–F bond (∼880 cm^–1^) (Table S7), and a weak signal of NaF (400 cm^–1^) emerged.^36,37^ Oppositely, the PTFE’s spectra exhibited a significant difference during the test (Figure 3e). The peaks in the green and red ranges are associated with C–F vibrations, which nearly disappeared after more than 3 days of reaction.^38^ Meanwhile, the C=C-related peaks in the orange region and C–OH-related signals in the blue area intensified considerably owing to the weakening of spectral features.^39^ Before normalization, the absorbance intensities were very low because the membrane turned completely black after 5 days of reaction, reducing IR reflectivity greatly. Beyond surface characterizations, chronoamperometry analysis was utilized to evaluate the reaction rate between these three polymers and Na metal by applying 0 V relative to their open-circuit voltage and tracking the self-discharge current in each cell for 24 h. In Figure 3f, the PTFE exhibited the highest self-discharge current, which means the fastest reaction rate with Na metal, followed by PVDF-HFP, and the PVDF had the slowest reaction rate. Additionally, changing the water content during the PVDF-HFP fabrication process might alter its effective contact area with Na and affect the reaction rate. However, compared to the influence of intrinsic properties of functional groups, this factor might have a smaller impact on the reaction rate.

Considering the formation of NaF, the presence of C=C bonds, and the reduction in pore size after degradation, it is suspected that one of the decomposition reactions that PFAS undergoes is similar to the Wurtz reaction mechanism.^200^ Such an alkyl halide coupling reaction is initiated by the Na radical attack to abstract fluorides, followed by further electron exchange between Na and carbon radicals to yield organosodium species (Na^+^-R^–^). The as-formed carbon anion (C^–^) can act as a nucleophile to attack other carbon atoms and extend the chain or as a base to trigger an elimination reaction and form C=C bonds.

### Density Functional Theory (DFT) Computational Studies

To illustrate the reaction mechanisms physicochemically, understand the distinct reaction rate phenomenon, and explore all potential reaction pathways of these three polymers, DFT calculations were performed at the B3LYP/6-31+G** level with the SMD solvation model for TEGDME using the Gaussian 16 package. For each polymer, a 12-carbon chain was defined and modeled to create a comparable electronic environment at the single-molecule level. Figure 4a shows the optimized geometries of the PVDF-HFP, PVDF, and PTFE molecules. The carbon chain was slightly twisted from a staggered to a gauche conformation to minimize ground-state energy under the effect of the explicit solvation model in each case. To first reveal reactive sites on each molecule, the Fukui function (*f* ^0^, Figure 4b) and the dual descriptor (Δ*f*, Figure S11) were computed by Multiwfn to examine molecular preferences toward Na radicals and carbon anions, respectively (see SI for detailed values, Figures S12–S14).^40–43^ For the PTFE chain, fluoro groups near the central region exhibited relatively higher *f* ^0^ values, indicating preferred sites for the Na radical attack. Meanwhile, dual descriptor analysis revealed that the carbons in the −CF_2_– groups are more electrophilic. In contrast, the *f* ^0^ positive isosurface in PVDF spread across both hydrogen and fluorine atoms, which reduces the *f* ^0^ values of fluorine sites. Also, the Δ*f* values of carbons were greatly suppressed due to the electron-donating effect of methylene groups within the molecule. Some carbon sites even transitioned from electrophilic to nucleophilic behaviors. After the HFP segment was introduced, the *f* ^0^ positive surface was more localized on the fluorides in the HFP units. The fluoro group next to the trifluoromethyl group showed the highest condensed *f* ^0^ value, followed by the −CF_2_– fluorides adjacent to it. The Δ*f* value also indicates that the carbon bonded to the trifluoromethyl group has the greatest chance for nucleophilic attack. Therefore, the Fukui function and dual descriptor analyses suggest that the central region of polymers has a higher reactivity preference for radical and nucleophilic attack steps in Wurtz reactions.

**Figure 4:**
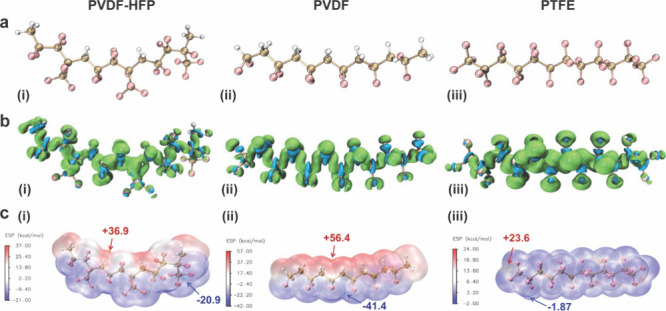
(i) PVDF-HFP, (ii) PVDF, and (iii) PTFE. (a) Optimized structures (pink: F, white: H, brown: C). (b) Computed Fukui functions (*f* ^0^) (isosurface = 0.001 au, green-preferred sites: *f* ^0^ > 0, blue-less preferred sites: *f* ^0^ < 0). (c) Electrostatic potential at van der Waals surface (isosurface = 0.001 au). Global surface maximum and minimum points were shown by yellow and cyan balls, respectively. Digit labels pointed out the surface energy of extreme points in kcal/mol.

Furthermore, the electrostatic potential (ESP) surface was computed using Multiwfn, as shown in Figure 4c, to facilitate a comparative analysis of reactivity between different polymers.^44^ Since PTFE consists solely of carbon and fluorine atoms, electrons are strongly withdrawn and dispersed by fluorides on the van der Waals ESP surface. Due to the large number of high electronegative fluorides present, the surface ESP value remains slightly below 0, with a global minimum of −1.82 kcal/mol, indicating that all fluorides in PTFE are under the most electron-deficient environment among the three polymer models. On the other hand, the ESP map of PVDF had a more negative potential around its fluorides with a global minimum of −41.4 kcal/mol. This high negative potential indicates the less reactive fluoro groups in PVDF, which also explains why there is only a small amount of NaF detected in the IR spectrum. Nevertheless, the positive ESP surface was exposed clearly since methylene protons point in the opposite direction of the −CF_2_– groups, resulting in a maximum potential of 56.4 kcal/mol. This unique structure and the most positive potential indicate high reactivity of the methylene groups toward C^–^ nucleophilic attack or elimination reaction, which explains why PVDF underwent rapid discoloration within 5 min after contact with Na metal. Last, PVDF-HFP incorporating a highly fluorinated HFP segment lowered the global minimum to −20.9 kcal/mol. In its structure, interestingly, methylene groups in the PVDF segment align in the same direction as the −CF_2_– groups in HFP segment, which produces a moderate potential surface with a global maximum of 36.9 kcal/mol. This periodical change from near 0 to slightly positive potential might be the reason that its color changes more gradually than the others when in contact with Na metal.

### Mechanisms and Byproducts between PFAS and Sodium Metal

Based on the above analyses, the PFAS degradation mechanisms and potential byproducts are proposed in Figure 5. It should be noted that only the most ready-to-react positions were drawn since there are numerous possible reacting sites. Consistent with the previously proposed Wurtz reaction, the degradation of all three polymers is initiated by the abstraction of fluoride by Na radicals. Instead of terminating after the first step, the resulting carbon radical can further react with the Na radical to produce a carbon anion, which can act as a nucleophile for nucleophilic attacks to induce cross-linking phenomena or act as a base for elimination reactions to generate C=C bonds. While cross-linking reactions could block membrane pores, the C=C bond formation could gradually establish a conjugation system to produce the dark color observed in the experiment. Specifically, PTFE requires an intramolecular biradical reaction to form C=C bonds (Figure 5c-i) due to the lack of protons.

**Figure 5:**
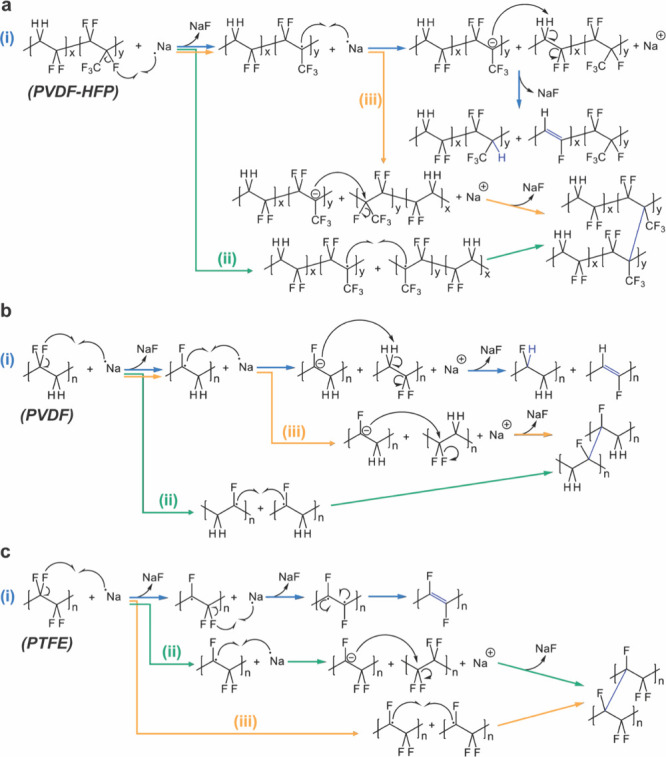
Proposed decomposition mechanism between PFAS polymers and Na metals: (a) PVDF-HFP reaction pathways, (b) PVDF reaction pathways, and (c) PTFE reaction pathways.

These reactions are unique to Na-based systems. The PVDF-HFP membrane had no observable color change when pressed with Li metal and soaked in the EC/DEC (1:1 wt %) solvent after 14 days (Figure S15c). This stability can be attributed to the intrinsic properties of Li metal and the denser packing structure of the Li-related SEI layer, which inhibits Li metal from further reacting with PFAS membranes.^45^ The solvent was changed from TEGDME to EC/DEC because Li metal reacts severely with TEGDME (Figures S15a and S15b). Moreover, the structure and composition of the solvents also influence these defluorination reactions. However, rather than directly affecting the inherent interactions between Na metal and PFAS membranes, the side reactions between solvents and Na metal might create a passivation layer and indirectly reduce the extent of contact between membranes and Na metal to slow down the overall reaction rate (Figure S16).

## Conclusions

In this study, we point out a previously overlooked and undesired degradation reaction occurring at the interface between polyfluoroalkyl-based polymers and Na metal. This work is the first paper to systematically analyze the mechanisms and byproducts of this degradation, providing a comprehensive analysis of how specific chemical structures affect the reaction rate of these side reactions. Through a series of experiments, we demonstrate that this phenomenon happens exclusively when the PVDF-HFP membrane comes into direct contact with Na metal and is independent of Na^+^ concentration and solvents. Moreover, similar reactions are also observed in PVDF and PTFE membranes, further underscoring the broader impact of this interfacial instability.

This chemical degradation induces polymer cross-linking and the formation of conjugated C=C bonds, leading to surface morphology changes and a darkened membrane color. By evaluating the reaction behaviors of PVDF-HFP, PVDF, and PTFE, we conclude two key strategies for designing PFAS membranes that are more compatible with Na batteries. The first method is enhancing the electron density of fluorides by increasing the hydrocarbon moiety to reduce the initial radical-driven reaction between fluoro groups and Na metal. The second strategy focuses on modifying the polymer chain structure or introducing sterically hindered functional groups to prevent excessive cross-linking and elimination reactions while still allowing for controlled reactivity for forming a NaF-rich interface. These insights provide valuable guidance for novel polymer membrane design and offer a pathway toward enhanced battery stability and performance in Na-based systems.

Lastly, although only a small part of Na batteries use Na metal as anodes, Na plating and stripping are common phenomena on electrodes across all types of Na batteries during cycling. Even if Na-ion intercalation compounds are used, metallic Na can still gradually form and accumulate in the localized regions. This “dead Na” may react with PFAS binders, potentially posing safety risks. Thus, these defluorination reactions are not exclusive to Na-metal batteries; they might happen in any Na-based systems. Beyond Na, similar reactions might also occur in potassium or other alloy systems if their ionization energies are low enough and can form radicals readily. However, further experiments are needed to validate this hypothesis.

## Experimental Section

### Materials

The PVDF-HFP pellet (average Mw ∼ 400,000, average Mn ∼ 130,000), PVDF membrane (0.22 μm pore size, hydrophobic, average thickness is 115 μm), sodium metal, sodium sulfide (Na_2_S, 98%), sulfur powder (S, 99.98% trace metals basis), and tetraethylene glycol dimethyl ether (TEGDME, ≥99%) were purchased from Sigma-Aldrich. The PTFE membrane (average thickness is 200 μm) was obtained from McMaster-Carr. Sodium perchlorate (NaClO_4_, 98.0–102.0%) was purchased from Fisher Scientific. Carbon paper (AvCarb P50, 0.007” thickness) was purchased from Graphite Store. Celgard 2400 was bought from Celgard. All the materials were used without further purification. Sodium polysulfide (Na_2_S_8_) was synthesized following our previous report.^46^

### Fabrication of the PVDF-HFP Membrane

All PVDF-HFP membranes (average thickness of 45 μm) in this study were prepared from PVDF-HFP pellets. The polymer solution was first prepared in acetone at 7.5 wt %, followed by adding 4.2 wt % of deionized H_2_O. The resultant solution was then ultrasonicated in a water bath for 30 min until all solids dissolved. The clear solution was poured on a glass plate and cast to a piece of membrane using a doctor blade with the casting gauge set to 0.5 mm. After the residue solvent was evaporated in air, the membrane was peeled off by applying ethanol between the membrane and the glass plate. Membranes were dried in a vacuum oven at 70 °C overnight and stored in an Ar-filled glovebox.

### Cell Assembly

1. Na||S Cells (PVDF-HFP). CR2032 coin cells were assembled in an argon-filled glovebox. The components were stacked in the following order: anode case, stainless steel (SS) spring, 0.5 mm SS spacer, sodium metal (12 mm diameter), PVDF-HFP membrane (15 mm diameter), carbon paper (10 mm diameter), and cathode case. During the assembly process, 20 μL of 1.0 M NaClO_4_ in TEGDME electrolyte was added on the surface of Na metal before stacking with the PVDF-HFP membrane, and 12 μL of catholyte (0.25 M Na_2_S_8_ + 1.0 M NaClO_4_ in TEGDME) was dispensed onto two sides of carbon paper.
2. Na||Na Symmetric Cells (PVDF-HFP and Celgard 2400). The components were stacked in the same order as the Na-Polysulfide cell except for replacing carbon paper/catholyte with Na metal (12 mm diameter) and adding 20 μL of 1.0 M NaClO_4_ electrolyte between the membrane and Na metal.
3. Na||SS Asymmetric Cells (PVDF-HFP, PVDF, and PTFE). For chronoamperometry testing. The core structure is arranged in the order of 0.5 mm SS spacer, Na metal (10 mm diameter), testing membrane, and 0.5 mm SS spacer. Moreover, 80 μL of 1.0 M NaClO_4_ electrolyte was added on both sides of the separators.

### Electrochemical Measurements

1. Battery Cycling Tests. The cycling performance was evaluated using a Landt battery testing system operating in galvanostatic mode. The Na||S cells were cycled between 1.2 and 2.8 V at a current density of 0.329 mA/cm^2^ and at a temperature of 25 °C (Supplementary Note 1). The charge/discharge procedure was performed 200 times. The Na||Na symmetric cells were cycled at 0.088 mA/cm^2^, with each cycle consisting of a 1-h charge and 1-h discharge steps and repeated for 200 cycles.
2. Electrochemical Impedance Spectroscopy Test (EIS). BioLogic potentiostat was used with the frequency range from 0.2 MHz to 0.1 Hz.
3. Chronoamperometric Analysis. Testing was conducted using BioLogic potentiostat. The applied voltage was set to 0.0 V relative to their open-circuit voltage (OCV).

### Separation and Cleaning Steps for Samples before Characterizations

Due to the soft texture of sodium and the fragility of soaked membranes. All tested samples for postcycling analysis were first immersed in anhydrous ethanol (>99.45%) inside the glovebox to separate them from Na metal. Right after separation, the samples were transferred to a fresh ethanol solvent and washed multiple times to thoroughly remove the residual Na metal, the TEGDME solvent, or the NaClO_4_ electrolyte. Finally, they were dried in the vacuum oven at 70 °C overnight before characterizations. More details about potential reactions during the cleaning process are given in Supplementary Note 2.

### Characterizations

The morphological analysis was carried out using scanning electron microscopy (SEM, Hitachi SU 8230), and 12 nm of Au/Pd was coated on the analyzed samples by a Quorum Q-150 V ES Plus coater before taking SEM images. Raman spectroscopies were measured with a Renishaw Raman Spectrometer (488 nm laser excitation). Nicolet 6700 FT-IR instrument was used for Fourier-transform infrared (FT-IR) absorbance spectroscopy. The X-ray photoelectron spectroscopy (XPS) was measured with a Thermo Scientific K-Alpha system. High-resolution C 1s, F 1s, O 1s, and Na 1s spectra were measured, and peaks were corrected by first fitting the carbon peak and then shifting it to the correct positions. Solid-state ^13^C, ^1^H, and ^19^F MAS-Nuclear magnetic resonance (NMR) was collected on a500 MHz Bruker AV3-NMR spectrometer at spinning rate from 22 to 24 kHz (Supplementary Note 3). DFT calculations (Supplementary Note 4).

## Supplementary Material



## Notes

Theoretical capacity of the Na||Na_2_S_8_ cell and its cycling performance; possible side-reactions might happen during the cleaning process; solid-state NMR operation process; DFT calculation process; Nyquist plot of the Na||Na_2_S_8_ cell; SEM of PVDF-HFP in the Na||Na_2_S_8_ cell; photos of PVDF-HFP under different conditions; SEM of PVDF-HFP under different conditions; XPS of Na 1s, O 1s, and survey scan of PVDF-HFP; ^19^F NMR under different spin rates; NMR spectrum of ^1^H; photos of PVDF and PTFE under different conditions; dual descriptor models of polymers; detailed Fukui functional value of polymers; photo of Li with PVDF-HFP under different conditions; detailed value of EIS results; detailed value of FT-IR data; detailed value of Raman data; and detailed value of XPS data (PDF)
