# Supplementary material for: Unveiling the Reactivity of Fluoropolymers with Sodium Metal: Mechanistic Insights and Battery Implications
Source: JACS Au. 2025 Jun 26;5(7):3513–20. doi: 10.1021/jacsau.5c00552 (PMC12308392; doi:10.1021/jacsau.5c00552)
Supplement: Supplementary file 1 [file au5c00552_si_001.pdf]

Supporting Information for

Unveiling the Reactivity of Fluoropolymers with Sodium Metal: Mechanistic Insights and Battery Implications

Cheng-Tien Hsieh[†,#], Wenda Wu[‡,#], Karam Eeso[†], Zhitao Chen[†], Johannes Leisen[§], Alexandros Filippas[†], Michelle Lehmann[‡], Guang Yang[*,‡], Nian Liu[*,†]

[†] School of Chemical and Biomolecular Engineering, Georgia Institute of Technology, Atlanta, Georgia 30332, United States

[‡] Chemical Science Division, Oak Ridge National Laboratory, Oak Ridge, Tennessee 37831, United States

[§] School of Chemistry and Biochemistry, Georgia Institute of Technology, Atlanta, Georgia 30332, United States

[#] These authors contributed equally.

* Correspondence: nian.liu@chbe.gatech.edu (N.L.), yangg@ornl.gov (G.Y.)

Table of Contents

Supplementary Notes 3

Supplementary Note 1. 3

Supplementary Note 2. 4

Supplementary Note 3. 5

Supplementary Note 4. 6

Supplementary Figures 7

Figure S1. 7

Figure S2. 8

Figure S3. 9

Figure S4. 10

Figure S5. 11

Figure S6. 12

Figure S7. 13

Figure S8. 13

Figure S9. 15

Figure S10. 16

Figure S11 17

Figure S12 18

Figure S13. 19

Figure S14 20

Figure S15. 21

Figure S16. 22

Supplementary Tables 23

Table S1 23

Table S2. 23

Table S3 23

Table S4. 23

Table S5. 23

Table S6. 24

Table S7. 24

Table S8. 25

DFT Computational data: 26

Reference 29

Supplementary Notes

Supplementary Note 1. The specific capacity of polysulfide catholyte (0.25 M Na_2S_8 + 1.0M $NaClO_4$ in TEGDME)[1].

Equation S1 and Equation S2 are the main reaction mechanisms of Na_2S_8 catholyte during the discharge process. Na_2S_5 has high solubility in TEGDME solvent. But the solubility of Na_2S_4 is low in TEGDME (<< 0.1M), which might cause some solid precipitation and irreversible capacity degradation.

$$Na_2S_8 + 2Na^+ \rightarrow 2Na_2S_4 \ldots Eq.S1$$

$$5Na_2S_8 + 6Na^+ \rightarrow 8Na_2S_5 \ldots Eq.S2$$

If using Equation 1 as an example to calculate the specific capacity of our Na-Polysulfide batteries.

$$C_{specific} = \frac{nF}{M_{Na_2S_8}} \cdot \frac{1}{3600} = \frac{2 \cdot 96485}{302.5 \cdot 3600} = 177.2\, mAh/g$$

The concentration of Na_2S_8 catholyte is 0.25M, and the volume is 12μL. The weight of Na_2S_8 solute would be $9.075 \times 10^{-4}\ g$. Thus, the ideal total capacity of our batteries is 0.1364 mAh. Some total capacity value and their corresponding specific capacity are as follows,

- 0.10 mAh → 110.2 mAh/g
- 0.15 mAh → 165.3 mAh/g

When Na_2S_8 only reduces to Na_2S_5, the specific capacity will decrease. However, if Na_2S_8 further reduces Na_2S_3 or even lower S ratio compounds, the specific capacity will increase in that cycle and permanently decrease the total capacity for the following cycles because of the loss of active materials. Thus, the fast degradation of the Na-Polysulfide battery's capacity is not just due to the separator's surface morphology change but also the material properties of our catholyte.

Supplementary Note 2. The possible reactions and by-products that might be generated during the cleaning process

The following mechanisms are the potential reactions that may occur during the ethanol cleaning process. It is important to note that PVDF-based polymers are susceptible to reacting with NaOH, leading to defluorination, which results in forming C=C bonds that could impact our experimental results. (Eq.S8)[2]. To avoid that situation, preventing Na metal from direct contact with water is necessary, and ethanol is a good solvent to remove residual Na metal that sticks to the membranes.

3. Na metal with ethanol:

$$2Na + 2C_2H_5OH \rightarrow 2C_2H_5ONa + H_2 ... Eq.S3$$

$* \, C_2H_5ONa$ *will dissolve in the ethanol.*

4. Na metal with air:

$$2Na + H_2O \rightarrow 2NaOH + H_2 \ ... Eq.S4$$

$$4Na + 2CO_2 + O_2 \rightarrow 2Na_2CO_3 \ ... Eq.S5$$

$$4Na + O_2 \rightarrow 2Na_2O \ ... Eq.S6$$

$$2Na + O_2 \rightarrow Na_2O_2 \ ... Eq.S7$$

5. PVDF-based polymers with NaOH[2]:

$$CH_2 - CF_2 \ + NaOH \rightarrow CH = CF + NaF + H_2O \ ... Eq.S8$$

Supplementary Note 3. Detailed parameters of solid-state NMR

For solid-state NMR studies a500 MHz Bruker AV3-NMR spectrometer (Bruker) operating with a dual channel MAS (magic angle spinning) probe for 3.2 o.d. MAS rotors was used. The maximum spinning rate achievable with this setup is 24 kHz. One channel of the probe can be tuned to ^{1}H or ^{19}F, the second channel is a broad-band channel, allowing tuning to a broad range of frequencies including ^{13}C.

^{19}F spectra were recorded at high spinning speeds of up to 24 kHz using simple Bloch decays and no ^{1}H decoupling. The repetition delay between scans was 5 sec and 128 averages were recorded for each spectrum.

^{1}H spectra were recorded for spinning speeds of 24 kHz using again simple Bloch decays with a recycle delay of 5 sec and 32 averages.

^{13}C spectra were recorded using the direct polarization of the ^{13}C nuclei with ^{1}H decoupling. The repetition delay between scans was 4 sec and 20,000 averages were recorded. For ^{1}H and ^{19}F nuclei it was confirmed that the repetition delay was selected long enough to achieve a complete T1-relaxation to ensure that peak intensities correspond to relative molar concentrations. The experimental conditions for the ^{13}C experiment do lead to a spectrum which enhances mobile non-crystalline moieties. A detailed study investigating structural differences between amorphous and crystalline regions using different decoupling and cross-polarization schemes is beyond the scope of this study.

Supplementary Note 4. The DFT computational methods[3-8]

Density functional theory (DFT) calculations for the three polymers were conducted using the Gaussian 16 package. Specifically, all polymers were defined to a 12-carbon chain model and computed at B3LYP/6-31+G** level with the SMD solvation model for TEGDME. SMD universal solvation model was applied to model the solvent environment using dielectric constants. True minimums were confirmed for all optimized geometries by verifying the absence of imaginary frequencies. All optimized geometries' coordinates are given on later pages. The tetraglyme solvation cavity was defined in the SMD solvation model with the following inputs:

```
#p B3LYP/6-31+G** opt freq=noraman pop=reg scrf(SMD,solvent=generic,read)
Symmetry=none

[Title]

[Charge] [multiplicity]
Coordinates

stoichiometry=C10O5H22
solventname=Tetraglyme
eps=7.79
epsinf=2.05
vmol=220.3
density=0.0027
```

Fukui function (f^0), dual descriptor (Δf), and electrostatic potential (ESP) surface were calculated by Multiwfn and visualized by VMD software[9].

Supplementary Figures

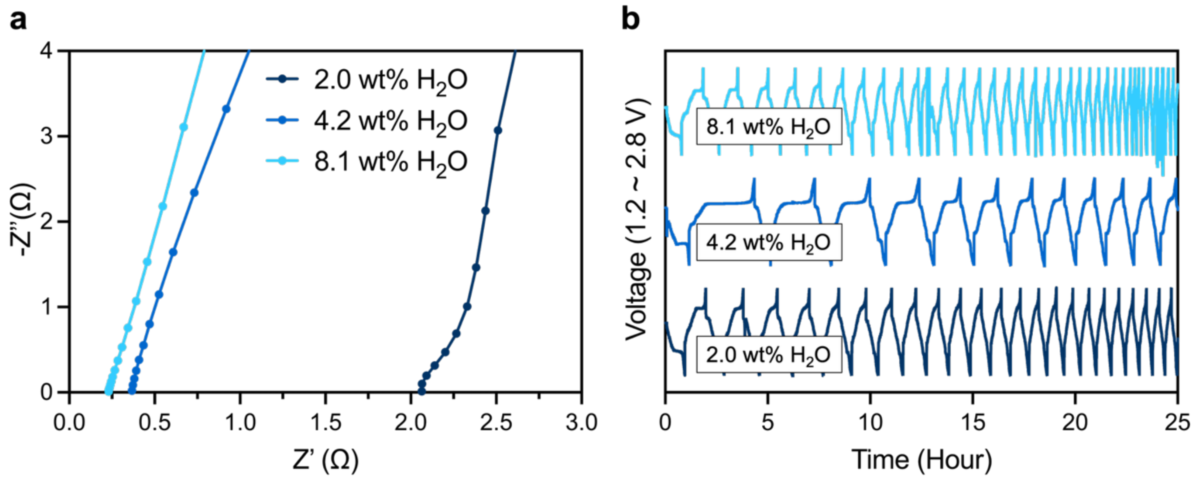

Figure S1. The performance of PVDF-HFP membranes with different water ratios. (a) Nyquist plots of PVDF-HFP membranes in Spacer||Spacer cell. (b) Electrochemical performance of PVDF-HFP membrane in Na||Na_2S_8 cells.

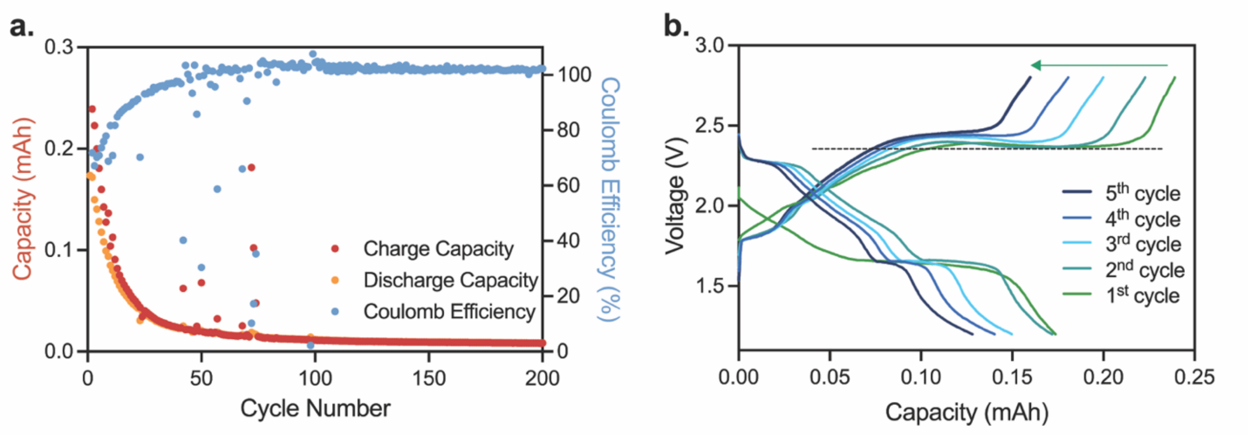

Figure S2. Electrochemical performance of Na-Polysulfide battery with PVDF-HFP separator. (a) Cycling performance (± 0.329 mA/cm^2). (b) Galvanostatic charge/discharge voltage profiles of first five cycles.

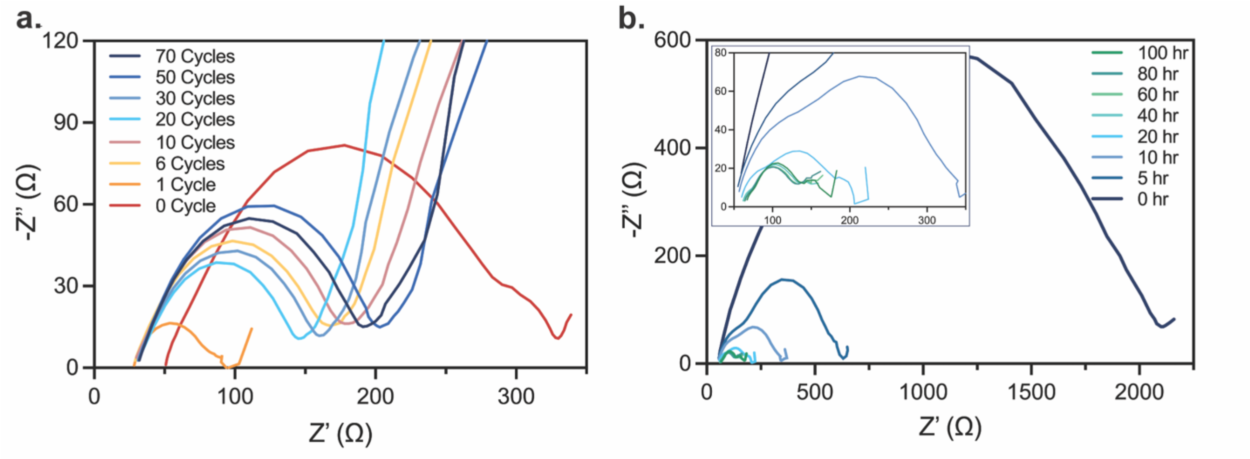

Figure S3. Nyquist plots of Na||Polysulfide cells with PVDF-HFP separator. (a) Under different cycle numbers. (b) Under different resting times without cycling. Insert: Magnified view of the same figure, highlighting the curves after ten hours of rest.

- *The detailed value of resistance can check Tables S1 and S2.*

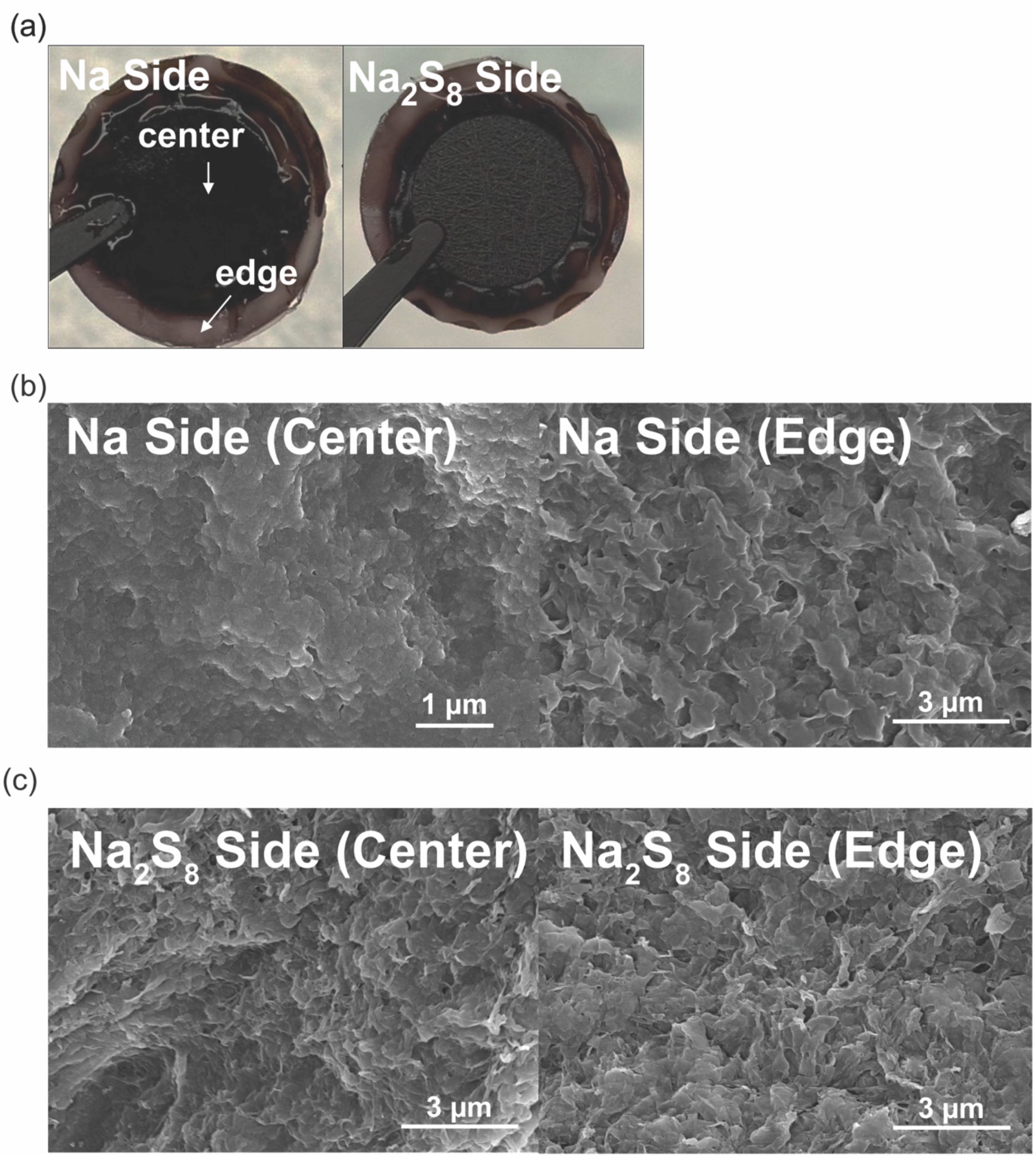

Figure S4. The PVDF-HFP separator's surface morphology in the Na-Polysulfide battery after finishing 200 cycles. (a) Photos of the membrane facing different electrodes. (b) SEM images of different areas on the side of the membrane facing the Na metal electrode. (c) SEM images of different areas on the side of the membrane facing the sodium polysulfide (Na_2S_8) electrode.

- *The central region refers to the area in direct contact with Na metal, whereas the edge section means the area not directly exposed to Na metal.*

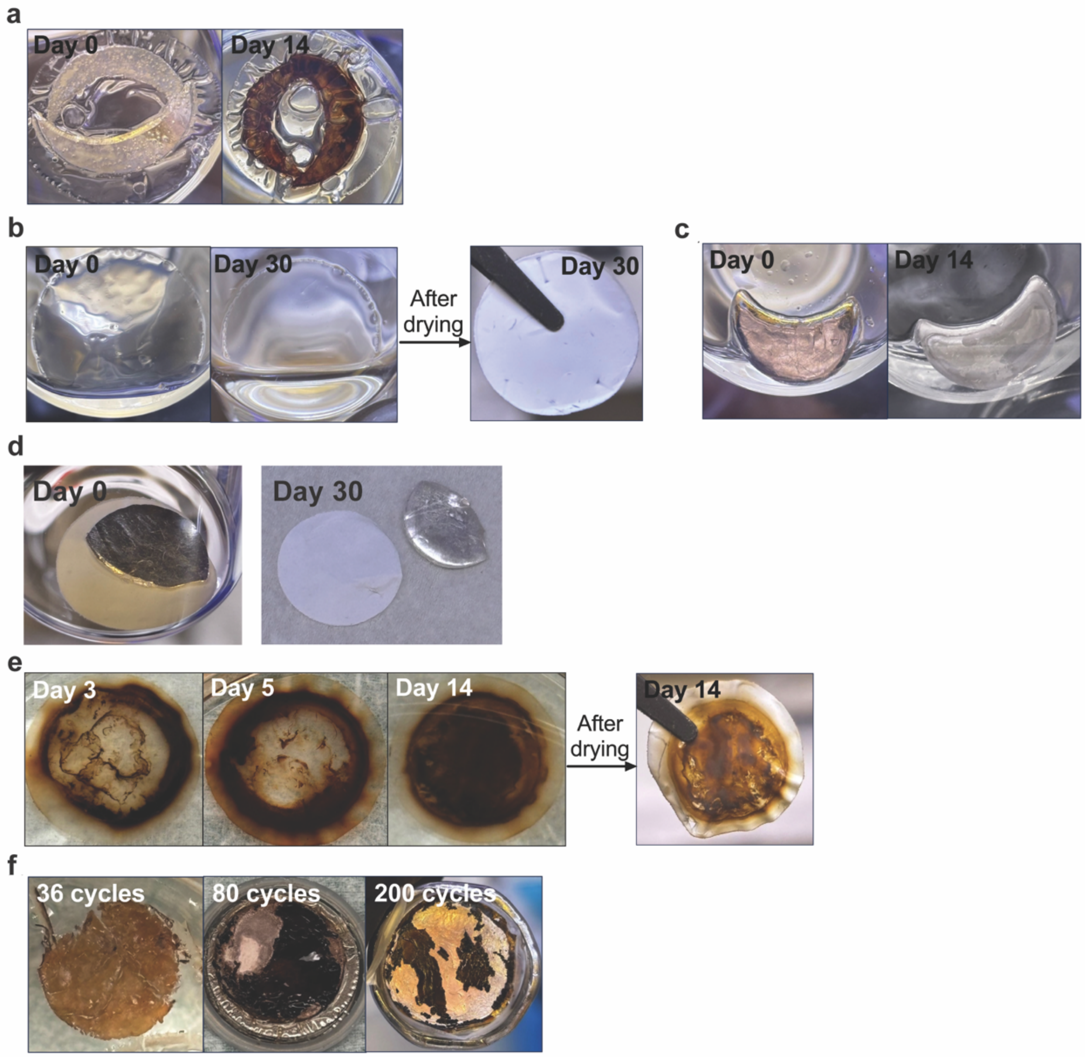

Figure S5. (a) The photographs of PVDF-HFP membranes after different reaction times with sodium metal in 1.0 M $NaClO_4$ electrolyte. (b) The pictures of PVDF-HFP membrane under different soaking times in 2.0 M $NaClO_4$ electrolyte. (c) The pictures of sodium metal under different soaking times in 1.0 M $NaClO_4$ electrolyte. (d) The photographs of Na metal and PVDF-HFP membrane at different reaction times without soaking in a solvent. (e) The photographs of PVDF-HFP membranes after different reaction times with sodium metal in TEGDME. (f) The photographs of PVDF-HFP membranes after different cycle numbers in the Na||Na symmetric cells.

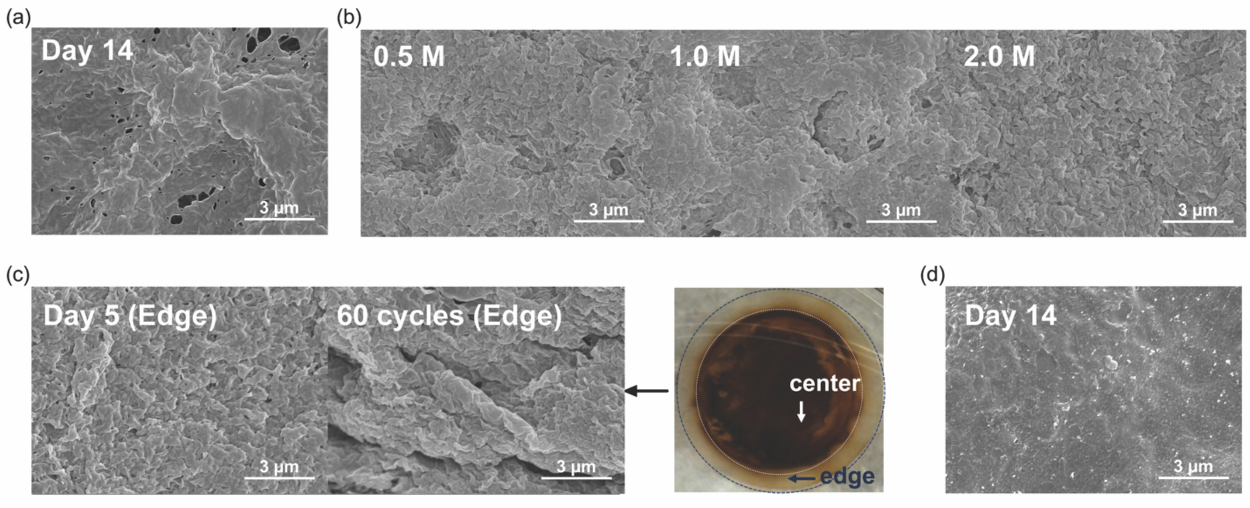

Figure S6. The SEM images of PVDF-HFP membranes. (a) The images of membrane after soaking in TEGDME for 14 days. (b) The images of membrane after soaking in different concentrations of $NaClO_4$ electrolyte for 30 days. (c) The images of the edge region of PVDF-HFP membrane under different reaction conditions. The left image is after reacting with sodium metal for 5 days in TEGDME solvent, and the right one is after cycling for 60 cycles in a Na-Na symmetric cell. (d) After reacting with sodium metal in TEGDME solvent for 14 days.

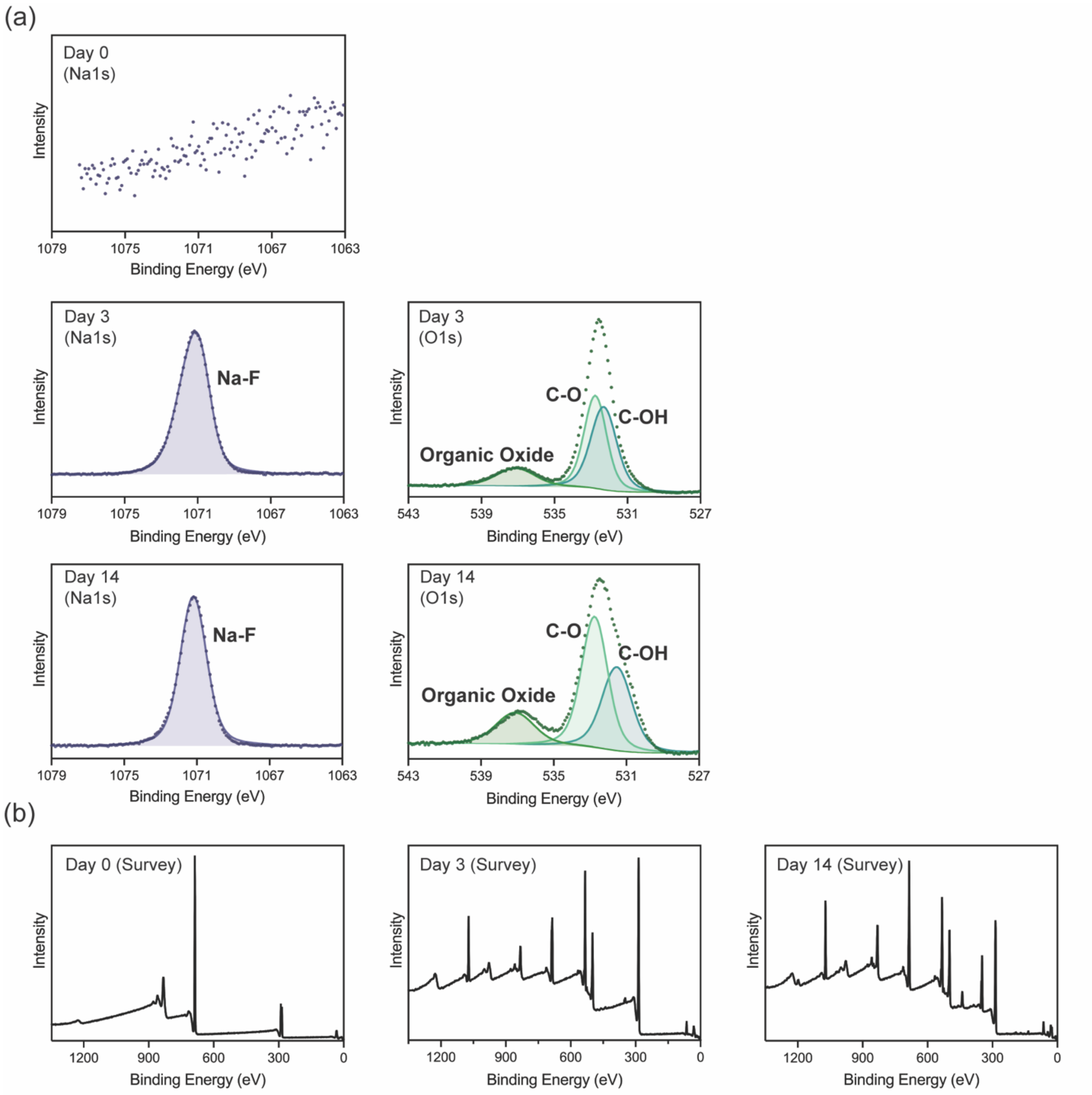

Figure S7. The XPS results of PVDF-HFP membranes under different reaction times with sodium metal. (a) The element scans (Na1s and O1s). (b) The survey scan.

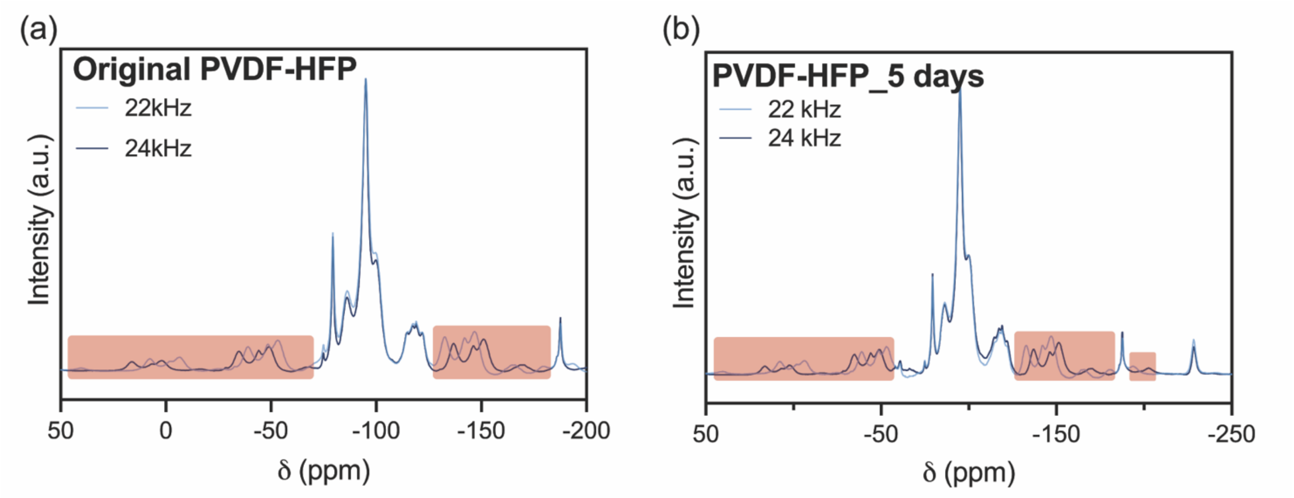

Figure S8. The ^{19}F NMR data of PVDF-HFP at different spin rates. The peaks inside the red region belong to spinning sidebands (SSBs) and are not real peaks. (a) Original PVDF-HFP membrane. (b) The PVDF-HFP membrane reacted with sodium metal for five days.

- *The SSBs peaks will shift under different spin rates (Hz). Comparing the data at different spin rates can distinguish the real and spinning sideband peaks.*
- *Peaks in the range of -115 ppm to -120 ppm are related to defect structures caused by irregular head-to-head or tail-to-tail monomer sequences in the amorphous domains.*

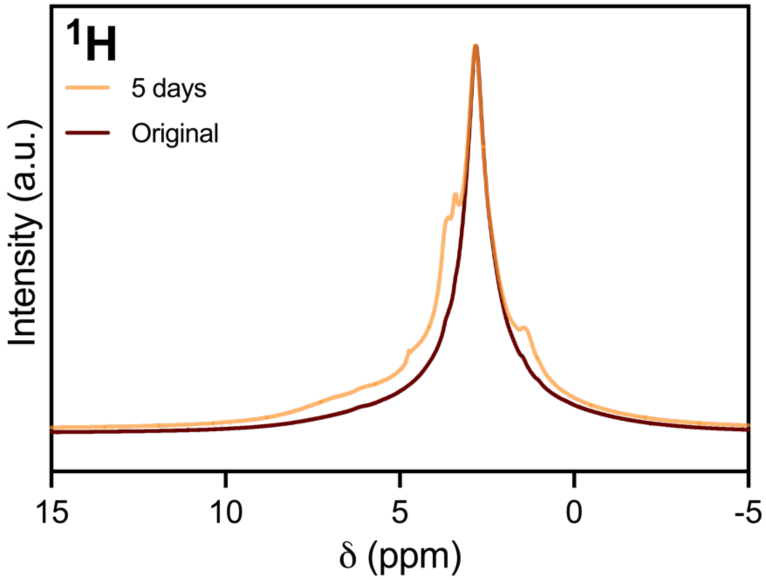

Figure S9. The normalized ^{1}H NMR data (24 kHz) of PVDF-HFP at different reaction times.

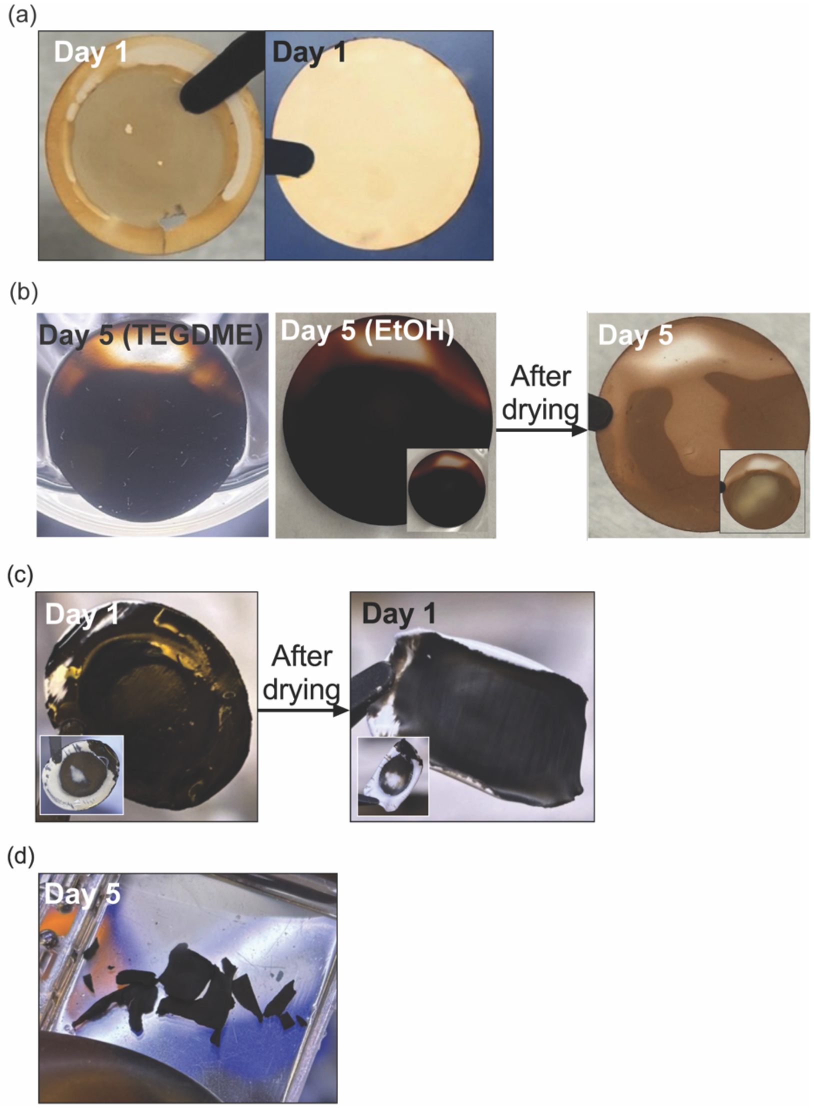

Figure S10. The pictures of membranes after different reaction times with Na metal. (a) Dried PVDF membrane after reacting 1 day with sodium metal. The left is the face that directly touches sodium metal, and the right is the side without contact. (b) PVDF membrane after reacted with Na metal for five days. From left to right: Soaking in the TEGDME solvent; Soaking in the ethanol; After drying. Insert: The side that didn't directly contact with sodium metal. (c) PTFE membrane reacted with Na metal for one day. The left one is soaked in the TEGDME solvent, and the right is after drying. Insert: The side that didn't directly contact with sodium metal. (d) The membrane fragments of PTFE membrane after reacting with sodium metal for five days.

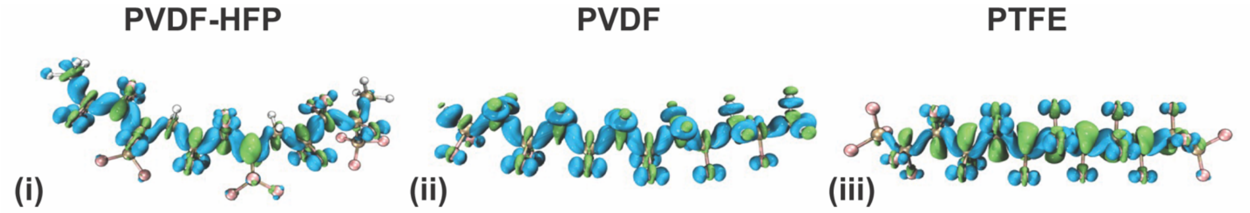

Figure S11. Computed dual descriptors (isosurface = 0.002a.u., green-electrophilicity sites: $\Delta f > 0$, blue-nucleophilicity sites: $\Delta f < 0$) for (i) PVDF-HFP, (ii) PVDF, and (iii) PTFE polymer chains.

- *Optimized structure: slightly twisted from staggered to gauge conformation to reduce the ground state energy under the effect of explicit solvation model.*

(a)

cFF for F in f^0

Atom	cFF
18(F)	0.0415
15(F)	0.0413
17(F)	0.0407
14(F)	0.0394
6(F)	0.0393
2(F)	0.039
11(F)	0.037
4(F)	0.0365
12(F)	0.0356
5(F)	0.0347
8(F)	0.0319
9(F)	0.0294
20(F)	0.0288
21(F)	0.0288
30(F)	0.0249
31(F)	0.0227
23(F)	0.0211
24(F)	0.0208
34(F)	0.018
33(F)	0.0161

(b)

cDD for C in Δf

Atom	cDD
13(C)	0.018
16(C)	0.0178
1(C)	0.0162
10(C)	0.0157
3(C)	0.0138
19(C)	0.0109
7(C)	0.0108
29(C)	0.0072
22(C)	0.0065
32(C)	0.0037
25(C)	0.0034
35(C)	0.0012

(c)

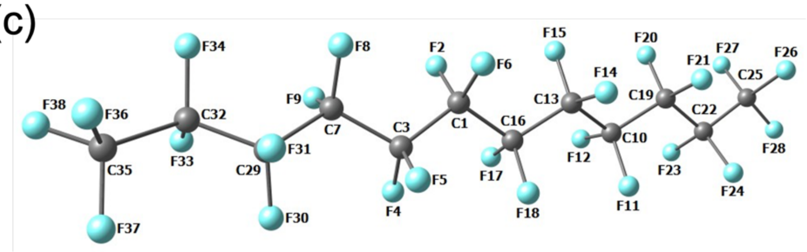

Figure S12. PTFE (a) Table of condensed Fukui function values for radical attack. (b) Table of condensed dual descriptor for nucleophilic attack. (c) Molecule structure. Terminal fluorides on –CF_3 were removed.

(a)

cFF for F in f^0

Atom	cFF
14(F)	0.0363
2(F)	0.0343
3(F)	0.0335
15(F)	0.0325
8(F)	0.0273
9(F)	0.0265
21(F)	0.0264
20(F)	0.026
33(F)	0.0168
34(F)	0.0163

(b)

cDD for C in Δf

Atom	CDD
10(C)	0.0119
16(C)	0.0018
4(C)	-0.0019
13(C)	-0.0089
22(C)	-0.0097
1(C)	-0.0104
19(C)	-0.0114
25(C)	-0.0124
32(C)	-0.0141
7(C)	-0.0146
29(C)	-0.0162

(c)

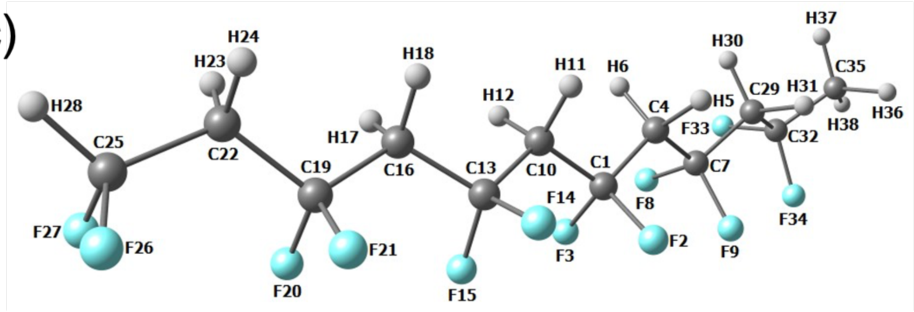

Figure S13. PVDF (a) Table of condensed Fukui function values for radical attack. (b) Table of condensed dual descriptor for nucleophilic attack. (c) Molecule structure. Terminal carbons (C35) were removed.

Although H has even higher cFF in f^0, Na is not strong enough to deprotonate alkyl fluoride (forming NaF is easier than forming NaH in the reaction).

(a)

cFF for F in f^0	
Atom	cFF
17(F)	0.0667
4(F)	0.0458
23(F)	0.0401
2(F)	0.0354
12(F)	0.0297
5(F)	0.0291
11(F)	0.0284
6(F)	0.0272
28(F)	0.0271
26(F)	0.025
25(F)	0.0222
29(F)	0.022
21(F)	0.0212
20(F)	0.0199
39(F)	0.0194
42(F)	0.0182
40(F)	0.0169
31(F)	0.0166
19(F)	0.0138
32(F)	0.0123
33(F)	0.0111
48(F)	0.0066
49(F)	0.0059
50(F)	0.0051

(b)

cDD for C in Δf	
Atom	cDD
16(C)	0.0262
18(C)	0.0242
30(C)	0.0113
1(C)	0.0068
22(C)	0.004
3(C)	0.0027
13(C)	0.0009
47(C)	0.0008
7(C)	-0.0022
10(C)	-0.0053
41(C)	-0.0095
24(C)	-0.0106
38(C)	-0.0133
27(C)	-0.0177

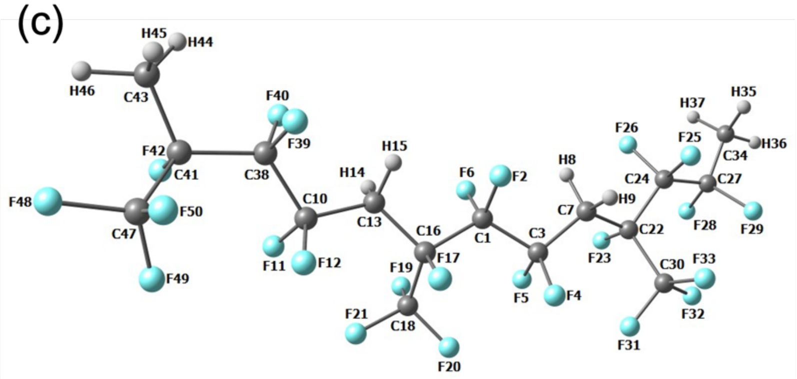

Figure S14. PVDF-HFP (a) Table of condensed Fukui function values for radical attack. (b) Table of condensed dual descriptor for nucleophilic attack. (c) Molecule structure. Terminal carbons (C43 and C34) were removed.

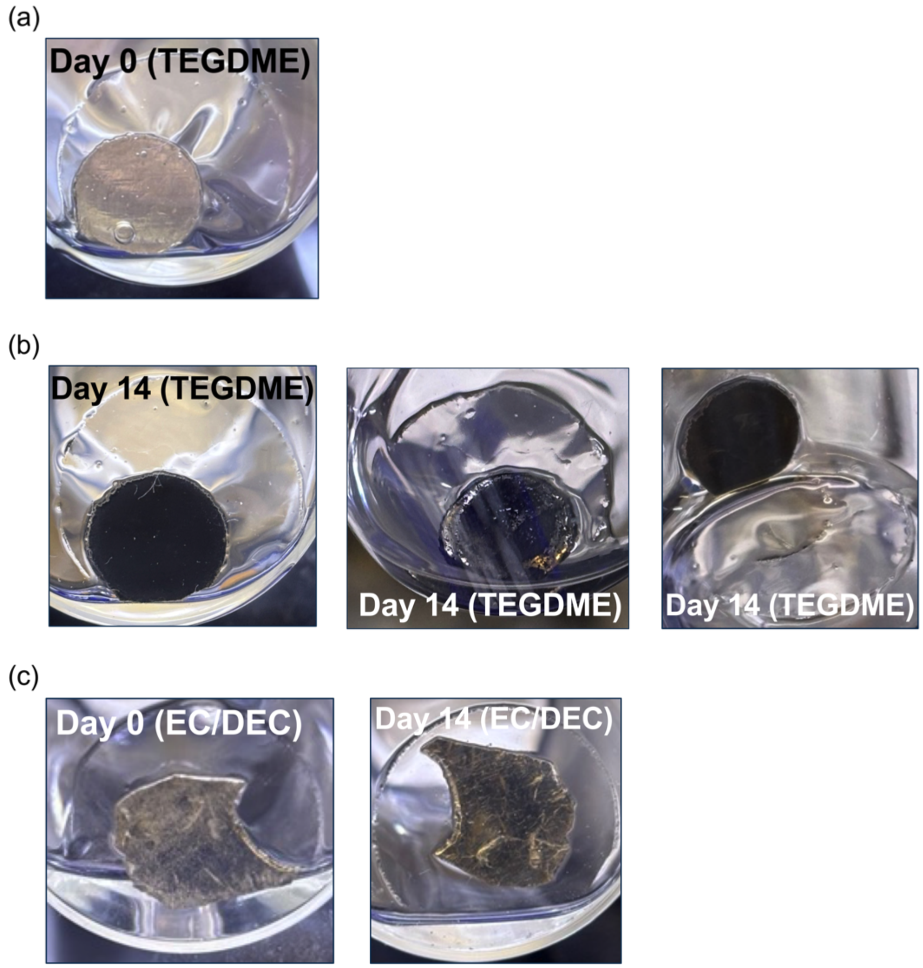

Figure S15. The pictures of PVDF-HFP membranes at different reaction times with lithium metal. (a) PVDF-HFP membrane and lithium metal were pressed together and soaked in the TEGDME solvent at day 0. (b) PVDF-HFP membrane and lithium metal were pressed together and soaked in the TEGDME solvent at day 14. The photographs from left to right: The side that directly comes into contact with the PVDF-HFP membrane; The side that didn’t encounter membrane; The picture when we separate the lithium metal (facing to membrane side) and membrane. (c) PVDF-HFP membrane and lithium metal soaked in EC/DEC (1:1 wt%) solvent.

- *It has a severe side reaction between lithium metal and TEGDME solvent.*

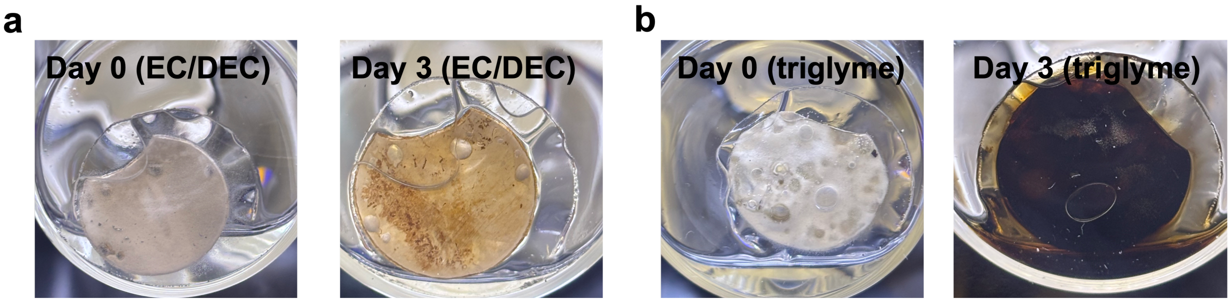

Figure S16. The photograph of Na metal and the PVDF-HFP membrane that were pressed and soaked in the different solvents (a) EC/DEC for 3 days. (b) Triethylene glycol dimethyl ether (triglyme) for 3 days.

Supplementary Tables

Table S1. The value of ohmic resistance and charge-transfer resistance presented in Figure S3a.

Cycle number	0	1	6	10	20	30	50	70
Ohmic resistance (Ω)	50.5	28.0	27.7	28.4	30.5	30.0	30.6	31.1
Charge-transfer resistance (Ω)	340	60	140	160	120	130	180	170

Table S2. The value of ohmic resistance and charge-transfer resistance presented in Figure S3b.

Reaction hour	0	5	10	20	40	60	80	100
Ohmic resistance (Ω)	54.8	51.8	53.6	58.1	60.5	62.0	62.8	65.4
Charge-transfer resistance (Ω)	2100	600	300	150	95	85	80	95

Table S3. The ohmic resistance value shown in Figure 1c.

Reaction hour	0	5	10	20	40	60	80	100
Ohmic resistance (Ω)	4.11	3.40	3.47	4.58	4.02	4.80	6.23	7.85

Table S4. The ionic conductivity (Ohmic resistance) of PVDF-HFP membranes in Figure 1c.
The average thickness of PVDF-HFP membrane: 45 μm; the effective area: 1.13 cm^2

Reaction hour	0	5	10	20	40	60	80	100
Ionic conductivity (S/cm)	9.69×10^{-4}	1.17×10^{-3}	1.15×10^{-3}	8.69×10^{-4}	9.91×10^{-4}	8.30×10^{-4}	6.39×10^{-4}	5.07×10^{-4}

Table S5. The ratio of C-F (1180 cm^{-1}) to C-CF_3 (1070 cm^{-1}) peaks in PVDF-HFP FT-IR Data (Figure 2a)

Reaction day	0	3	5	14
Ratio Value	1.82	1.11	0.88	0.68

Table S6. The Ratio of G-band to D-band in PVDF-HFP Raman Data (Figure 2b).

Reaction day	0	3	5	14
G-band/D-band Value	-	1.57	1.53	1.40

Table S7. The ratio of C-C (1184 cm^{-1}) to C-F (878 cm^{-1}) peaks in PVDF FT-IR Data (Figure 3d)

Reaction day	0	1	3	5
Ratio Value	0.870	0.916	0.917	1.04

Table S8. The Information of PVDF-HFP XPS Data's Peak Positions. (Figures 2c, 2d, and S7)

C1s Scan	C-CF_3	C-F	C-O/	C-C	C=C
Day 0 (Fresh)	291.9 eV	289.2 eV	287.0 eV	284.8 eV	-
Day 3	290.3 eV	286.4 eV	286.0 eV	284.8 eV	284.2 eV
Day 14	289.3 eV	286.2 eV	285.7 eV	284.8 eV	284.2 eV

F1s Scan	C-CF_3	C-F	Na-F
Day 0 (Fresh)	686.8 eV	685.9 eV	-
Day 3	687.2 eV	684.9 eV	683.9 eV
Day 14	686.9 eV	684.6 eV	683.9 eV

O1s Scan	C-O	C-OH	Organic Oxide
Day 0 (Fresh)	-	-	-
Day 3	532.7 eV	532.4 eV	537.1 eV
Day 14	532.6 eV	531.4 eV	537.3 eV

Na1s Scan	Na-F
Day 0 (Fresh)	-
Day 3	1071.1 eV
Day 14	1071.2 eV

DFT Computational data:

Coordinates of optimized geometry (Figure 4a)

PTFE model molecule				PVDF model molecule			
Atom	X	Y	Z	Atom	X	Y	Z
C	0.681	3.389	-5.101	C	-1.054	0.721	-0.075
F	1.681	4.108	-5.665	F	-0.088	1.584	0.418
C	0.706	1.93	-5.693	F	-2.264	1.266	0.324
F	1.692	1.226	-5.088	C	-0.863	-0.663	0.546
F	-0.482	1.342	-5.42	H	0.19	-0.931	0.419
F	-0.503	3.956	-5.432	H	-1.457	-1.387	-0.019
C	0.951	1.884	-7.247	C	-1.236	-0.828	2.02
F	0.212	2.855	-7.836	F	-2.614	-0.76	2.156
F	2.262	2.13	-7.479	F	-0.746	0.236	2.761
C	0.866	4.978	-1.384	C	-1	0.674	-1.603
F	0.343	3.95	-0.671	H	-0.155	0.048	-1.903
F	2.216	4.922	-1.315	H	-1.914	0.179	-1.94
C	0.429	4.801	-2.885	C	-0.863	2.009	-2.339
F	-0.92	4.872	-2.952	F	0.41	2.519	-2.123
F	0.963	5.817	-3.608	F	-1.727	2.946	-1.804
C	0.866	3.434	-3.536	C	-1.106	1.858	-3.841
F	2.171	3.199	-3.27	H	-2.072	1.364	-3.982
F	0.122	2.448	-2.979	H	-0.333	1.191	-4.235
C	0.419	6.337	-0.723	C	-1.124	3.129	-4.691
F	1.128	7.334	-1.306	F	-2.21	3.913	-4.332
F	-0.898	6.55	-0.95	F	-0.01	3.908	-4.422
C	0.646	6.399	0.83	C	-1.179	2.816	-6.185
F	1.908	6.002	1.121	H	-1.987	2.1	-6.366
F	-0.228	5.555	1.429	H	-0.24	2.325	-6.461
C	0.442	7.811	1.477	C	-1.387	3.972	-7.146
F	-0.781	8.29	1.21	F	-0.461	4.98	-6.957
F	1.359	8.683	1.037	F	-2.631	4.557	-6.983
F	0.574	7.688	2.807	H	-1.314	3.65	-8.187
C	0.568	0.509	-7.908	C	-0.709	-2.141	2.6
F	0.999	-0.501	-7.114	H	-0.922	-2.945	1.889
F	-0.782	0.441	-7.999	H	0.378	-2.049	2.674
C	1.183	0.299	-9.337	C	-1.246	-2.587	3.958
F	2.496	-0.001	-9.203	F	-2.595	-2.924	3.835
F	1.067	1.445	-10.051	F	-1.228	-1.525	4.857

C	0.522	-0.845	-10.177	C	-0.515	-3.758	4.57
F	-0.722	-0.513	-10.545	H	0.527	-3.49	4.761
F	0.48	-1.988	-9.474	H	-0.546	-4.615	3.891
F	1.255	-1.046	-11.282	H	-0.998	-4.028	5.513

PVDF-HFP model molecule			
Atom	X	Y	Z
C	2.37	-1.293	-4.035
F	3.633	-1.057	-4.507
C	1.852	-2.58	-4.768
F	2.463	-3.659	-4.19
F	0.512	-2.675	-4.519
F	1.579	-0.26	-4.447
C	2.154	-2.508	-6.27
H	2.012	-1.471	-6.581
H	3.212	-2.747	-6.397
C	3.332	0.553	-0.673
F	2.235	0.845	0.092
F	3.995	-0.476	-0.065
C	2.93	0.212	-2.114
H	2.138	0.905	-2.403
H	3.794	0.418	-2.748
C	2.492	-1.232	-2.457
F	3.493	-2.13	-2.118
C	1.222	-1.72	-1.697
F	0.143	-0.992	-2.047
F	0.962	-3.015	-1.944
F	1.395	-1.606	-0.368
C	1.316	-3.356	-7.256
F	-0.024	-3.01	-7.156
C	1.771	-2.927	-8.712
F	3.132	-3.036	-8.786
F	1.464	-1.596	-8.812
C	1.196	-3.611	-9.995
F	-0.163	-3.75	-9.837
F	1.724	-4.883	-10.047
C	1.362	-4.892	-7.001
F	0.985	-5.174	-5.741
F	0.522	-5.557	-7.811

F	2.608	-5.375	-7.185
C	1.502	-2.881	-11.277
H	2.58	-2.755	-11.396
H	1.117	-3.49	-12.1
H	1.013	-1.906	-11.289
C	4.278	1.801	-0.631
F	5.445	1.436	-1.241
F	3.691	2.76	-1.414
C	4.632	2.511	0.724
F	3.444	3.083	1.186
C	5.653	3.615	0.48
H	5.328	4.244	-0.351
H	6.636	3.197	0.252
H	5.726	4.228	1.38
C	5.099	1.566	1.867
F	5.528	2.295	2.921
F	4.111	0.774	2.317
F	6.124	0.785	1.469

Reference

(1) Self, E. C.; Tyler, J. L.; Nanda, J. Ambient temperature sodium polysulfide catholyte for nonaqueous redox flow batteries. *Journal of the Electrochemical Society* **2021**, *168* (8), 080540.

(2) Ross, G.; Watts, J.; Hill, M.; Morrissey, P. Surface modification of poly (vinylidene fluoride) by alkaline treatment Part 2. Process modification by the use of phase transfer catalysts. *Polymer* **2001**, *42* (2), 403-413.

(3) Frisch, M.; Trucks, G.; Schlegel, H.; Scuseria, G.; Robb, M.; Cheeseman, J.; Scalmani, G.; Barone, V.; Petersson, G.; Nakatsuji, H. Gaussian 16, Revision C. 01. Gaussian, Inc., Wallingford CT. 2016. *There is no corresponding record for this reference* **2016**.

(4) Marenich, A. V.; Cramer, C. J.; Truhlar, D. G. Universal solvation model based on solute electron density and on a continuum model of the solvent defined by the bulk dielectric constant and atomic surface tensions. *The Journal of Physical Chemistry B* **2009**, *113* (18), 6378-6396.

(5) Petersson, a.; Bennett, A.; Tensfeldt, T. G.; Al-Laham, M. A.; Shirley, W. A.; Mantzaris, J. A complete basis set model chemistry. I. The total energies of closed-shell atoms and hydrides of the first-row elements. *The Journal of chemical physics* **1988**, *89* (4), 2193-2218.

(6) Petersson, G.; Al-Laham, M. A. A complete basis set model chemistry. II. Open-shell systems and the total energies of the first-row atoms. *The Journal of chemical physics* **1991**, *94* (9), 6081-6090.

(7) Becke, A. Density-functional thermochemistry. III. The role of exact exchange (1993) J. *Chem. Phys 98*, 5648.

(8) Stephens, P. J.; Devlin, F. J.; Chabalowski, C. F.; Frisch, M. J. Ab initio calculation of vibrational absorption and circular dichroism spectra using density functional force fields. *The Journal of physical chemistry* **1994**, *98* (45), 11623-11627.

(9) Humphrey, W.; Dalke, A.; Schulten, K. VMD: visual molecular dynamics. *Journal of molecular graphics* **1996**, *14* (1), 33-38.
